# Development of an elastic cell culture substrate for a novel uniaxial tensile strain bioreactor

**DOI:** 10.1002/jbm.a.34917

**Published:** 2013-08-31

**Authors:** Matthew D Moles, Colin A Scotchford, Alastair Campbell Ritchie

**Affiliations:** Division of Materials, Mechanics and Structures, Faculty of Mechanical, Materials and Manufacturing Engineering, University of Nottingham, University ParkNottingham, NG7 2RD, United Kingdom

**Keywords:** adhesion, bioreactor, osteoblast, polyurethane, surface modification, strain

## Abstract

Bioreactors can be used for mechanical conditioning and to investigate the mechanobiology of cells *in vitro*. In this study a polyurethane (PU), Chronoflex AL, was evaluated for use as a flexible cell culture substrate in a novel bioreactor capable of imparting cyclic uniaxial tensile strain to cells. PU membranes were plasma etched, across a range of operating parameters, in oxygen. Contact angle analysis and X-ray photoelectron spectroscopy showed increases in wettability and surface oxygen were related to both etching power and duration. Atomic force microscopy demonstrated that surface roughness decreased after etching at 20 W but was increased at higher powers. The etching parameters, 20 W 40 s, produced membranes with high surface oxygen content (21%), a contact angle of 66° ± 7° and reduced topographical features. Etching and protein conditioning membranes facilitated attachment, and growth to confluence within 3 days, of MG-63 osteoblasts. After 2 days with uniaxial strain (1%, 30 cycles/min, 1500 cycles/day), cellular alignment was observed perpendicular to the principal strain axis, and found to increase after 24 h. The results indicate that the membrane supports culture and strain transmission to adhered cells. © 2013 Wiley Periodicals, Inc. J Biomed Mater Res Part A: 102A: 2356–2364, 2014.

## INTRODUCTION

Typically *in vitro* culture involves the use of tissue culture polystyrene (TCPS) culture vessels. Cells are grown on the stiff (tensile modulus = 3 GPa^1^) substrate, usually without dynamic mechanical stimuli, whereas nutrient, gas, and metabolite exchange are limited by diffusion. Cells cultured *in vitro* often undergo dedifferentiation and therefore exhibit a phenotype not representative of the *in vivo* cell.^2^ The mechanical environment to which a cell is exposed is known to affect proliferation,^3^ migration^4^ and differentiation *in vitro* and *in vivo*.^5^,^6^ Tissue mechanics are dependent upon the cells present, the extracellular matrix (ECM) and the interactions between each cell and the ECM, mediated by integrins.^7^ The mechanical nature and dynamics of tissues vary widely; bone tissue has a relatively stiff tensile modulus (*ca*. 15–30 GPa), and under strenuous exercise bone has been shown to experience strains of 2000–4000 µstrain.^8^ With *in vitro* testing it has been reported that strains of 10 times greater magnitude (1–3% or 10,000–30,000 µstrain) are required to achieve an observable effect.^9^

**FIGURE 1:**
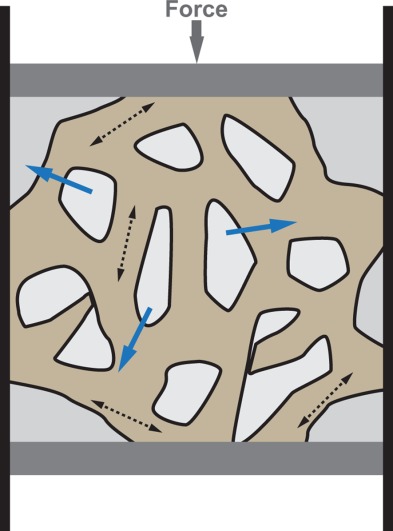
Diagram showing complex state of strain (dotted arrows) and fluid shear stress (solid arrows) in a porous scaffold under compression. [Color figure can be viewed in the online issue, which is available at http://wileyonlinelibrary.com.]

The use of synthetic and naturally derived substrates to facilitate induction of mechanical forces on cells *in vitro* using bioreactors has long been established.^10^,^11^ While the effects upon cells are well characterized, many methodologies may unintentionally expose cells to a combination of mechanical stimuli. For example, compression methods apply a compressive stress or strain by squeezing the substrate between two plates, as shown schematically in Figure 1. When stress is applied to a porous substrate, the compression will induce fluid flow because of the displacement of the culture medium within the substrate, and the scaffold will experience a combination of inhomogeneous compressive, tensile and shear stresses that are difficult to quantify, again because of the porous (and often random) architecture of the scaffold.^12^

**FIGURE 2:**
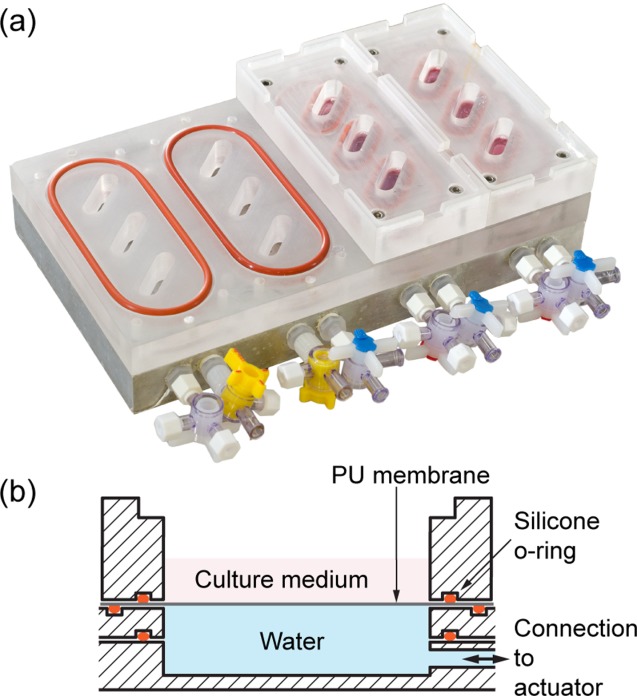
The bioreactor, (a), is shown with two assembled banks, each containing three culture wells. A cross-sectional diagram, (b), through a culture well shows the arrangement of the PU membrane. [Color figure can be viewed in the online issue, which is available at http://wileyonlinelibrary.com.]

The Flexcell system (Flexcell International, Hillsborough, NC) is often used in application of tensile biaxial strain to cells on a flexible substrate, but because of the actuation method, also applies significant fluid shear stress.^13^ Presented in this paper is the optimization of a candidate flexible membrane for direct strain application to cells cultured in a novel bioreactor (Fig. 2). Membranes are clamped under straight-edged oval-shaped wells that under positive volumetric displacement exert a small perpendicular stress (Equation 1) to overcome the perpendicular strain arising from Poisson's ratio, and thus give uniaxial strain (Equation 2). A polyurethane (PU) polymer, Chronoflex AL 80A, was selected as it fulfils the requirements for a highly elastic (strain at yield > 400%) and hydrolytic degradation resistant membrane.^14^ Many polymers have surfaces that are not conducive to cell attachment, and as the majority of mammalian cells are attachment dependent, the culture surface requires modification. Studies have shown that modifying surface hydrophilicity, chemistry and roughness, and incubating biomaterials with extracellular matrix proteins, such as fibronectin, have an enhancing effect on cell attachment.^15^,^16^ Surface modification of biomaterials has been accomplished using methods such gamma irradiation, ultraviolet irradiation, and plasma etching.^17^, ^18^ Plasma etching was selected for membrane optimization in this study as it is an effective method to induce stable changes to surface properties without significantly affecting the bulk material^17^:


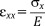
(1)


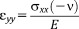
(2)

## MATERIALS AND METHODS

Reagents and materials were obtained from Sigma Aldrich (UK), and cell culture equipment, medium, and supplements from Gibco Invitrogen (UK) unless otherwise stated.

### Preparation of the bioreactor

A novel uniaxial tensile bioreactor developed in-house (Fig. 2) was used for experiments involving cell culture with a dynamic mechanical environment. A programmable microcontroller utilized a high-precision linear actuator to produce volumetric displacement. Uniaxial tensile strain, with a peak in the range 0.2–3.0%, can be induced in the substrate cyclically at up to 60 cycles per minute.

Prior to use, the bioreactor was cleaned with Trigene detergent (Medichem International, UK) diluted 1:10, before thoroughly rinsing with hot water and then double-rinsing and wiping with 70% ethanol. Plasma-etched PU membranes were inserted to complete the assembly. Each well was re-sterilized with 70% ethanol before washing with phosphate buffered saline (PBS), as the membranes were exposed to a non-sterile environment during transition after plasma-treatment. To improve cell attachment to the PU membrane it was protein conditioned^15^ for 48 h by incubating with culture medium containing 10% foetal bovine serum (FBS). A port in the side of the incubator allowed connection of the actuation system to the bioreactor via high-pressure (6 MPa) Luer lock tubing.

### Preparation of membranes

The preparation method for PU membranes is based upon that of Ritchie et. al.,^19^ in brief: PU membranes of 200 ± 10 (SD) μm thickness were prepared by solution-casting oven-dried (24 h, 50°C) pelleted Chronoflex AL 80A (AdvanSource Biomaterials, Wilmington, MA) at 10% (w/w) in tetrahydrofuran (99.9%, BHT-stabilized) on a covered float-glass mould. Membranes were air-dried under ambient conditions (22°C, 60% RH) for approximately 12 h and then removed from the glass while solvent remained on the glass interfacing side, as this has been shown to improve optical quality.^20^ Residual solvent was extracted by a vacuum-oven at 50°C for a minimum of 48 h. Membranes were handled at their edges by tweezers and stored in aluminium foil in an air-tight container to minimize contamination. Sample discs (11 mm diameter) were punched from three different membrane batches.

Plasma etching of the membranes was achieved using a RF glow discharge plasma barrel reactor (Biorad PT-7100, Biorad Laboratories, USA). The chamber was roughed out to 6 Pa, flushed with oxygen (99.5%, BOC, Surrey, UK) and maintained at 20 ± 2 Pa for 5 min, before activating the RF source. The forward powers were 20, 50, 100, and 180 W, whereas reflected power was <1, <3, <5, and <10 W, respectively. Oxygen flow continued for 1 min after terminating the RF source. Etched membranes were analyzed or used for culture within 3 h.

### Cell culture and assays

Culture medium was composed of DMEM supplemented with 10% FBS (Fisher Scientific, UK), 1% 200 m*M* l-glutamine, 5% 100× antibiotics-antimycotics, 5% 1*M* HEPES, 1% 100× MEM nonessential amino acids and 0.15 mg mL^−1^ l-ascorbic acid (Fisher Scientific, UK). MG-63 cells (European Collection of Cell Cultures) were cultured in a humidified 37°C, 5% CO_2_ incubator. Cells were passaged at approximately 80% confluence; they were harvested using 0.25% trypsin-EDTA. Passages 24–26 were harvested, using 0.25% trypsin-EDTA, for seeding.

Metabolic activity was determined by alamarBlue (AbD Serotec, Oxford, UK) diluted 1:10 in Hank's Balanced Salt Solution: cell cultures were washed with warm PBS and then incubated with 500 µL diluted reagent for 45 min. Eluted reagent was protected from light while gently shaken on a plate reader for 5 min. The fluorescence (ex. 560 nm, em. 590 nm) of a triplicate of 100 µL aliquots was evaluated using a fluorescence platereader (FLx800, Biotek Instruments, USA) and blanked against nonreduced reagent.

Static experiments were conducted on (a) TCPS in the form of as-received multiwell culture plates (Nunclon Delta, Fisher Scientific, UK); (b) plasma-etched discs of PU membrane were used as the culture substrate in multi-well culture plates; and (c) as-fabricated discs of PU membrane also placed into well plates. The cell growth area of PU static control samples was constrained to match the bioreactor well area (2.10 cm^2^) by utilizing ethanol-sterilized PTFE cylinders (manufactured in-house) secured by steam-sterilized high-vacuum silicone grease (Dow Corning, Barry, UK).^21^

Dynamic experiments applied cyclic uniaxial tensile strain of 1% at 30 cycles per minute for 1500 cycles; stimulation time points are specified in the results.

### Microscopy

Phase-contrast microscopy (Nikon FS-100 with Nikkor 10×, 20×, and 40× objective lenses, Nikon, Japan) was used to obtain images of live cultures. For fluorescence imaging the samples were fixed with 4% paraformaldehyde, permeabilized using 0.5% Triton-X100 in saline buffer (20 m*M* HEPES, 300 m*M* sucrose, 50 m*M* NaCl, 3 m*M* MgCl_2_, pH 7.6, 5 min), stained with Hoescht 33342 (5 µg mL^−1^ in DI water, 15 min) and FITC conjugated phalloidin buffered (1:100) with 1% bovine serum albumin in PBS. A microscope (Leica DMLB with epifluorescence attachment) and camera (Nikon DXM1200, Nikon, Japan) was used to capture the images. Cellular alignment was determined by measuring the angle of the long axis of individual cells in relation to the principal strain axis of a minimum of 100 fusiform cells across six fields of view for each timepoint. There were three samples per timepoint.

### Surface characterization

Atomic force microscopy (AFM) of PU membrane was performed within 3 h of plasma etching on a Dimension 3100 Scanning Probe Microscope with Nanoscope III controller (Digital Instruments, Santa Barbara, CA) in tapping mode at RT in air. Multi75 tips (Bruker Nano Surfaces) with nominal 3 N m^−1^ spring constant and 75 kHz resonance were used. Roughness measurements were obtained from height maps using the software Gwyddion, version 2.25.^22^

Contact angles (CAs) were measured by the sessile drop method using 5 μL droplets of distilled water; images were captured, approximately 30 s after applying the droplet, using a Nikon D7000 D-SLR with a Nikkor 105-mm micro lens (Nikon Imaging, Japan) and were manually analyzed using the angle function of ImageJ.^23^ Measurements were taken before and after washing the samples with 70% ethanol and PBS, as is carried out with bioreactor membranes.

X-ray photoelectron spectroscopy (XPS) spectra were recorded using an Axis Ultra (Kratos Analytical, Kyoto, Japan) with monochromated Al K_α_ (1486.6 eV) radiation source at a pass energy of 80 eV for survey spectra and 20 eV for high-resolution spectra. Two samples were taken from separate batches; three areas (700 × 300 µm) per sample (11 mm diameter) were analyzed. Spectra were analyzed using CasaXPS 2.3.16-pr1.4 (Casa Software, UK); saturated C was charge corrected to 285.0 eV.

### Statistics

Mean data is shown for a minimum of three repeats. One-way ANOVA followed by Dunnett's multiple comparisons test was performed using GraphPad Prism version 6.0b for Mac OSX (GraphPad Software, CA, USA). Statistical difference is indicated as follows: *P < 0.05; **P < 0.01; ***P < 0.001; ****P < 0.0001.

## RESULTS

### PU membrane surface modification and characterization

**FIGURE 3:**
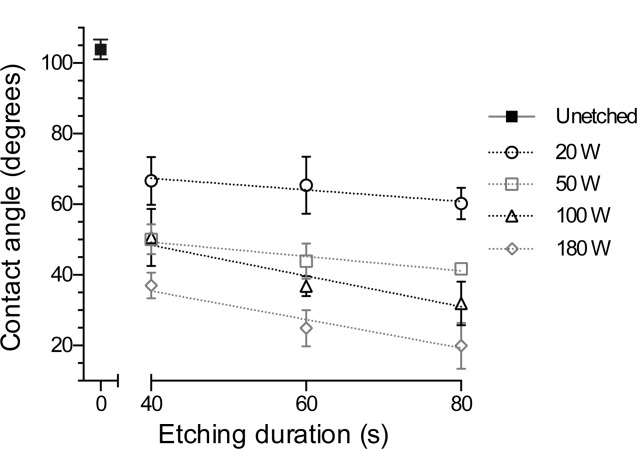
Contact angles for unetched PU, with weighted least square linear fit for plasma etched PU membranes. Data show mean ±SD for *n* = 3.

The wettability of the PU membrane was found to display a trend of decreasing CA related to both plasma power and plasma duration (Fig. 3). The highest CA was recorded for the relatively hydrophobic control surface, whereas the lowest angles were achieved by etching at the highest power, 180 W. The 20 W 40 s etching parameters produced a low CA that does not fit the trend. A least squares weighted linear fit was applied, which showed a strengthening relationship with etching power (*r*^2^ = 0.15, 0.49, 0.60, and 0.63 for 20, 50, 100, and 180 W). Washing the samples with ethanol followed by PBS increased the CA of etched samples; however, it decreased the CA of the unetched sample (shown in Supporting Information).

**FIGURE 4:**
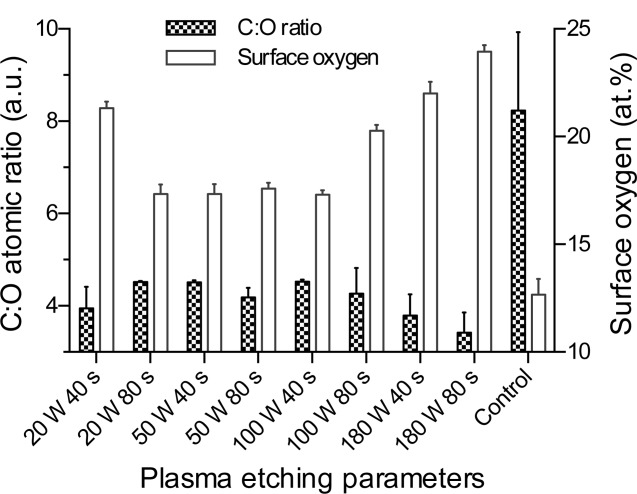
Surface oxygen and C:O ratio of as-fabricated control and plasma-etched PU samples, as determined by XPS. Data show mean ±SD for *n* = 6.

**FIGURE 5:**
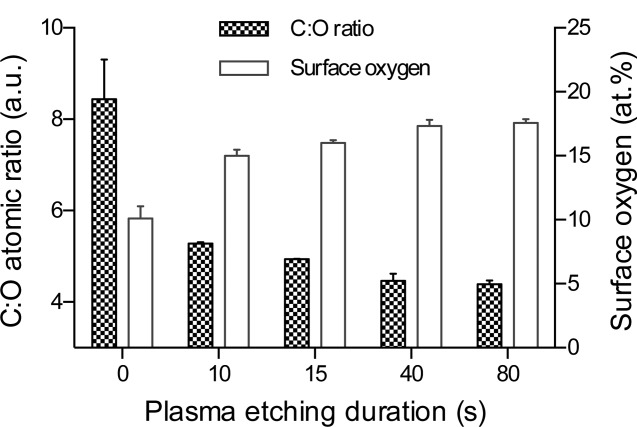
Relative atomic ratios, for carbon, oxygen, and nitrogen as determined by XPS, of PU membrane that was plasma etched at 50 W. Data show mean ±SD for *n* = 6.

Quantification of the surface elemental composition by XPS showed that carbon, oxygen, nitrogen, and silicon were present on all samples (shown in Supporting Information). It also confirmed that plasma treatment increased surface oxidation, as shown by the change in intensity of the O1s peak relative to the C1s peak. Lowest oxidation took place at 20 W 80s, 50 W 40 s, and 100 W 40 s (17.27 ± 0.45, 17.24 ± 0.45, and 17.18 ± 0.20 atomic% (at %), respectively), and the greatest oxidation was achieved at 180 W 80 s (23.95 ± 0.30 at %) as shown in Figure 4. Further XPS analysis of samples etched for shorter durations (Fig. 5) showed that the majority of oxidation was occurring within the first 10 s, and approached a plateau at between 15 and 40 s.

**FIGURE 6:**
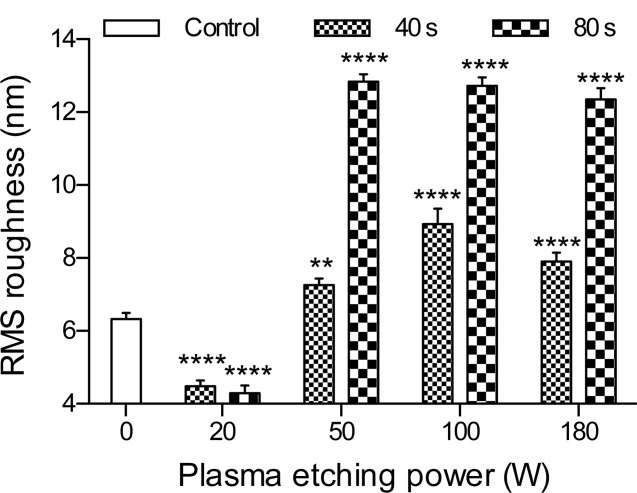
RMS roughness (Rq) of PU across a 25 µm sampling length. Data show mean with SEM for *n* = 20, and statistical significance with respect to the unetched sample is indicated.

**FIGURE 7:**
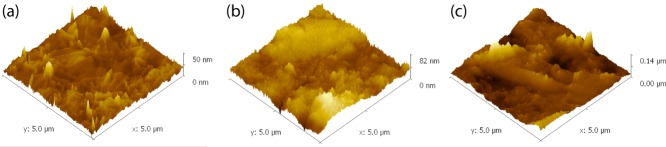
AFM height maps for: (a) unetched PU, and PU etched for 40 s at (b) 20 W and (c) 50 W. [Color figure can be viewed in the online issue, which is available at http://wileyonlinelibrary.com.]

Whilst SEM detected no difference in the PU topography, AFM showed a change in roughness following etching. Surface roughness (Fig. 6), as determined from the AFM height maps (Fig. 7), decreased after etching at 20 W with no statistically significant difference between 40 and 80 s etching durations. Etching for all other parameters increased the surface roughness. The as-fabricated surface had a segmented topography that after etching became nonsegmented and had a stippled appearance; this change occurred for all plasma etching parameters.

The effect of etching on protein adsorption was assessed by XPS after incubation of the high surface oxygen, low roughness membrane (20 W 40 s) with FBS-supplemented culture medium. The atomic percentage of sulphur was 0.29 ± 0.34 and 1.63 ± 0.61 at % for unetched and etched PU, respectively, after 2 days, whereas after 5 days these values increased to 1.26 ± 0.38 and 1.83 ± 0.62 at %; a statistical difference (*P* < 0.05) between etched and unetched samples was found for analysis at 2 days but not at 5 days. Silicon signals from the PU were not significantly attenuated on the unetched PU but were reduced to background levels on the etched PU (Supporting Information, Fig. SD 3).

As the purpose of modification was to increase cell adhesion through the addition of oxygen functionalities as opposed to topographical cues, the etching parameters of 40 s at 20 and 50 W, producing high and low increases in oxygen, respectively, were selected for *in vitro* evaluations.

### *In* *vitro* assessment of PU surface modification

**FIGURE 8:**
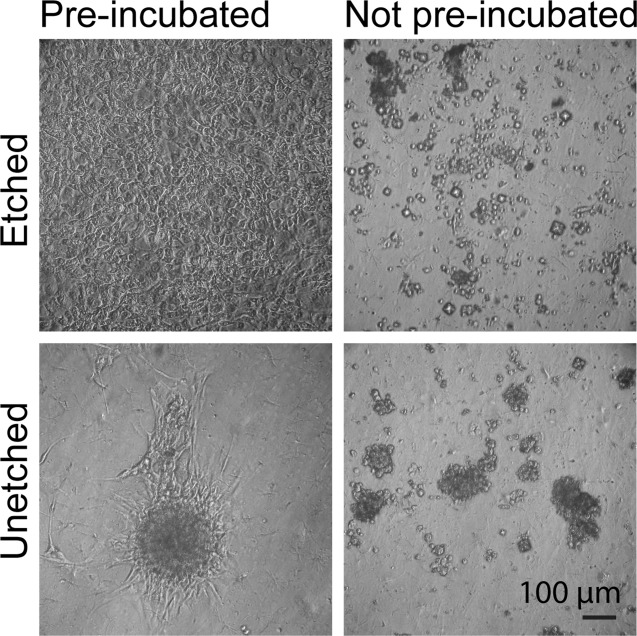
Phase contrast micrographs of MG-63 cells on pre-incubated and non pre-incubated PU substrates at 8 days after seeding. Etching was carried out at 20 W for 40 s.

When seeding directly onto etched and as-fabricated membranes, the cells had a round morphology, exhibited poor adherence to the substrate and were easily washed off during culture (Fig. 8). Distribution of cells on the etched PU was uniform but on the unetched PU the cells aggregated. Following the protein conditioning surface optimization step, confluent monolayers formed within 3 days on etched membranes, whereas on the as-fabricated unetched membranes cells remained in clusters although with some fusiform cells at the edges of the clusters. No difference in the cell morphology or latency to confluence was observed between PU etched at 20 or 50 W (40 s).

**FIGURE 9:**
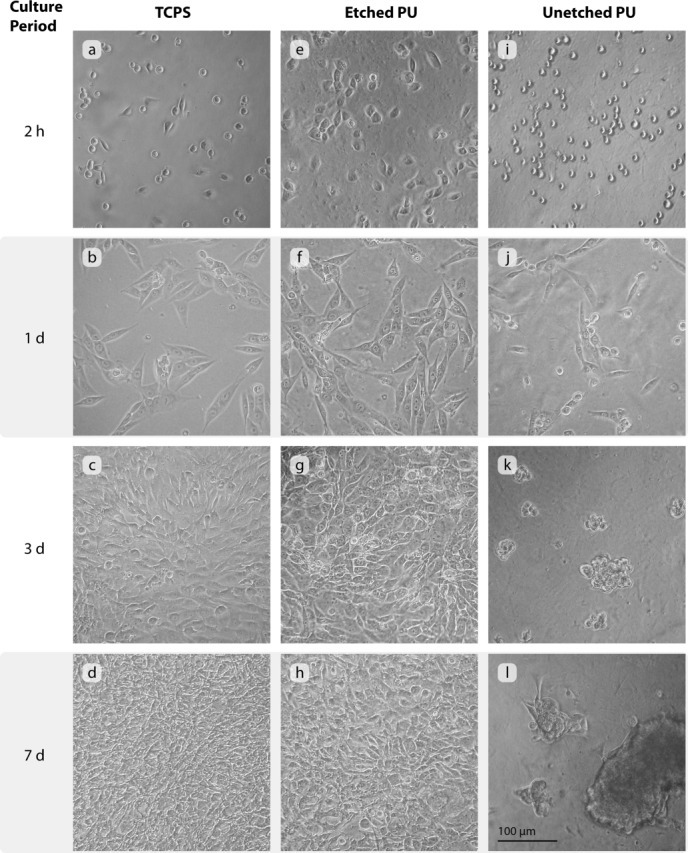
MG-63 cultures imaged by phase-contrast microscopy at selected intervals after seeding on (a)–(d) TCPS, (e)–(h) plasma-etched (50 W, 40 s) PU, and (i)–(l) unetched PU.

The morphology of MG-63 cultures seeded on TCPS, etched (50 W 40 s) and unetched PU was compared at the intervals of 2 h, and 1, 3, and 7 days after seeding at densities of 1 × 10^4^ and 2 × 10^4^ cells cm^−2^. Cells were found to attach poorly in the case of unetched PU at the lower seeding density. When seeding at 2 × 10^4^ cells cm^−2^ large clusters of adherent cells were formed, however when seeded at 1 × 10^4^ cells cm^−2^ the clumps were easily washed off the membrane during medium changes, and within 4 days of seeding relatively few cells remained. When seeding at 2 × 10^4^ cells cm^−2^ it was observed that 2 h after seeding, the morphology of the cells on the unetched PU [ Fig. 9(i)] was rounded and indistinguishable from that of cells on TCPS [ Fig. 9(a)], whereas on etched PU [ Fig. 9(e)] the cells had already begun to spread. After 1 day, cells on TCPS and etched PU had showed increased numbers and assumed fusiform morphologies [ Fig. 9(b,f)]; on unetched PU [Fig. 9(j)] the cells exhibited a mixture of rounded and fusiform morphology. TCPS and etched PU substrates achieved confluence within 3 days. However, on unetched PU the many small cell clusters that were observed at day 3 had aggregated into several larger clusters after 7 days; at 21 days the cells remained in large clusters.

The 20 W 40 s etching parameter was selected for further (dynamic strain) experiments as the smoother surface has fewer topographical features in comparison to the surface etched at 50 W.

### Dynamic cell culture

**FIGURE 10:**
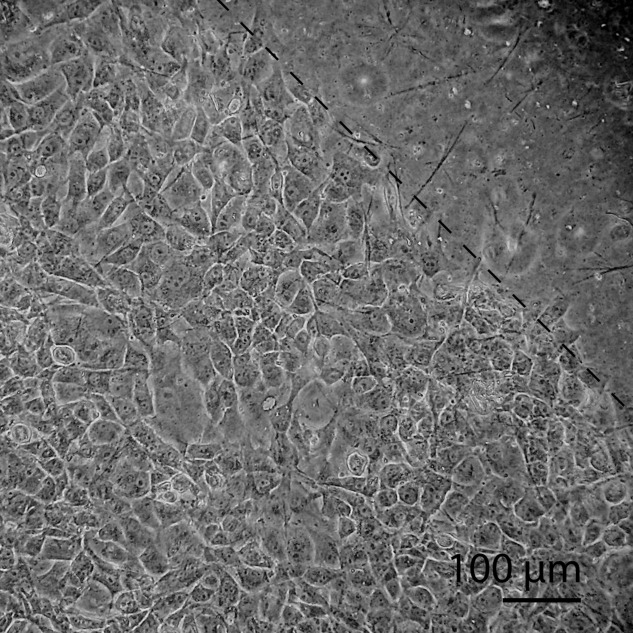
Phase contrast micrograph of MG-63s after 8 days culture in the bioreactor. The monolayer extends to the edge of the well (indicated by dashed line) where they are in contact with the food grade silicone o-ring.

**FIGURE 11:**
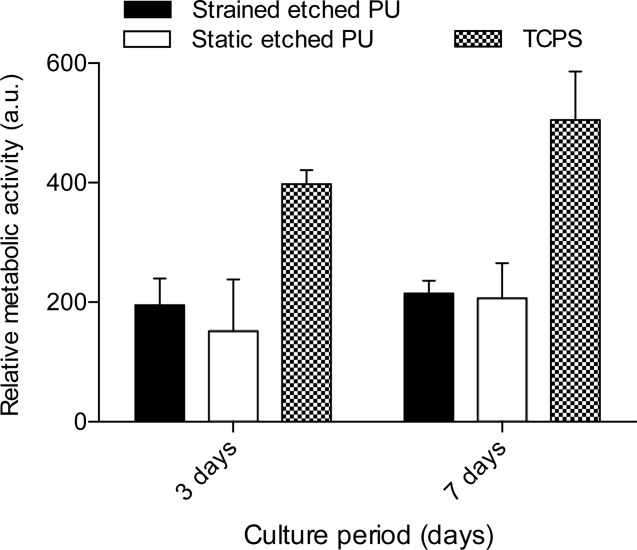
Relative metabolic activity of MG-63s after cyclic stimulation in the bioreactor. Data show mean ±SD for *n* = 6; TCPS vs PU: *P* < 0.0001.

Cyclic uniaxial tensile strain was applied to MG-63 cells seeded in the bioreactor at 2 × 10^4^ cells cm^−2^, beginning 24 h after seeding, for a period of 45 min each day. Confluent monolayers formed in the wells and cells remained attached as a monolayer after stimulation (Fig. 10). The metabolic activity (Fig. 11) of the cells after stimulation compared to unstimulated PU controls was not statistically significantly different; the activity, stimulated or unstimulated, was statistically significantly lower than for that of TCPS.

**FIGURE 12:**
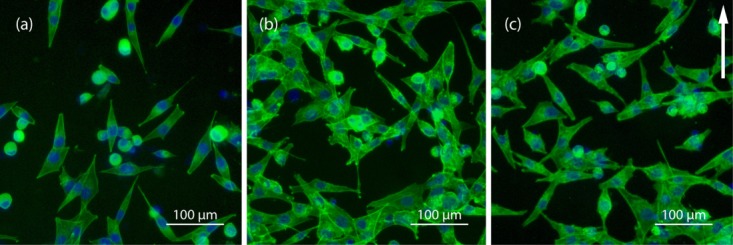
MG-63s stained for f actin (green) and DNA (blue). (a) Fixed 6 h after initial strain; (b) 6 h after second strain application; and (c) 24 h after second strain application. The principal strain axis is indicated by the arrow. [Color figure can be viewed in the online issue, which is available at http://wileyonlinelibrary.com.]

**FIGURE 13:**
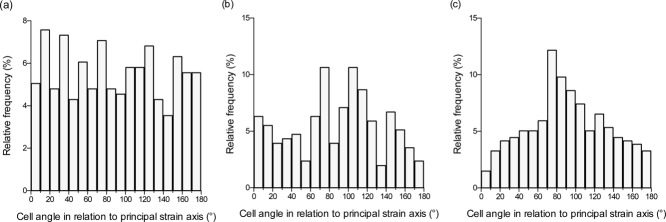
Alignment of cells relative to the principal strain axis: (a) 6 h after strain initial strain and (b) 6 h after second strain application at 48 h; and (c) 24 h after second strain application.

Cell morphological changes in response to strain were also evaluated. Samples were fixed 6 and 24 h after strain application (24 and 48 h), and then processed for fluorescence microscopy. Six hours after the initial strain application [ Fig. 12(a)] most cells displayed a fusiform morphology and alignment was random [ Fig. 13(a)]. When fixed 6 h after the second strain application [ Fig. 12(b)] some cells appeared to have spread and flattened, whereas at 24 h [ Fig. 12(c)] increased alignment, perpendicular to the principal strain axis, was seen [ Fig. 13(c)].

## DISCUSSION

Simulating the dynamic *in vivo* mechanical environment may provide further knowledge of cell responses to mechanical stimuli, and therefore could be used to drive specific responses that, for example, are of use to tissue engineering. A novel bioreactor capable of producing cyclic uniaxial tensile strain has been evaluated. The candidate elastic substrate, Chronoflex AL 80A polycarbonate urethane, was surface optimized for cell adhesion, and MG-63 cells were used to assess cell adhesion to the PU membrane and delivery of strain to the cells.

Initial cultures on as-fabricated PU membranes demonstrated poor cell adhesion; therefore, surface modification by plasma etching was carried out. The effects of oxidative plasma etching include surface ablation, surface cross-linking, and modification of the surface chemical composition to increase the quantity of oxygen containing groups.^17^,^24^ The presence of oxygen containing groups, specifically hydroxyl groups, on the surface increases wettability and is directly linked to cytocompatibility, cell adhesion, and growth.^25^–^27^ Moreover, binding of adhesion-mediating proteins is favored.^28^

CA analysis (Fig. 3) of PU showed that the surface hydrophilicity had a relationship with both the etching power and duration. TCPS CAs with deionized water are in the range 45–72,^29^–^32^ indicating that PU etched at 20 W 40 s has a CA (66° ± 7°) more suitable for *in vitro* cell culture. Inhibition of cell adhesion and proliferation may occur on highly hydrophobic (CA > 100°, because of change of the protein conformation) or hydrophilic (CA < 35°, because of the relatively weak binding of proteins) surfaces.^[^28,33,34 Delayed cell detachment, at 7 days, has also occurred on very hydrophilic substrates.^35^

Surface composition analysis by XPS (Fig. 4) showed that etching PU at 20 W 40 s samples resulted in 21.3 at % oxygen, which is closer to results for the etching parameters 100 W 80 s (20.3 at %) and 180 W 80 s (24.0 at %) than 20 W 80 s (17.3 at %) or unetched (12.7 at %). Samples etched for 40 s at 50, 100, and 180 W had a higher C:O ratio than for the corresponding 80 s samples, indicating less surface modification occurred, which correlates with the CA data.

AFM (Fig. 6) demonstrated a decrease in roughness following etching at 20 W but an increase for other etching powers. After 80 s etching at 50, 100, or 180 W the samples were significantly rougher, however, etching power was not a factor in the difference. The topography, initially consisting of crystal-like segments, had an amorphous appearance after etching. Fibers present at the surface of TCPS have been implicated in cell alignment^32^ therefore etching at 20 W 40 s, which was shown to reduce the surface roughness, is important to avoid confusing mechanostimulatory effects with roughness or topography-induced effects.

On both etched and unetched PU the MG-63 cells adhered poorly, and had a rounded appearance (Fig. 8). In a nonaqueous environment, such as air, PU displays hydrophobic domains on its surface, and in an aqueous environment the surface is rearranged to show hydrophilic segments in 25 h.^36^,^37^ This indicates that structural change may likely take place upon exposure of dry PU to culture medium, thus reducing or preventing protein absorption and cell attachment. Quantification of adhered proteins on PU incubated with culture medium for 2 days found a significantly greater proportion of proteins on etched compared to unetched PU. The detection of silicon after 2 and 5 days conditioning on as-fabricated membranes implied that the protein layer was less than 10-nm thick, whereas on the etched membrane the protein layer obscured the silicon. Serum contains a variety of adhesion and nonadhesion mediating proteins with different binding affinities,^34^ which may have led to a different complement of proteins on each surface type, and therefore account for the differences in cell morphology and metabolic activity. Protein conditioning promoted formation of confluent monolayers on the etched PU (Fig. 8). The results indicate that the surface chemistry, as opposed to the topography, is a critical factor in protein and cell adhesion and activity on PU.

MG-63 cells cultured on TCPS and pre-incubated PU are shown in Figure 9. On the unetched PU the cell morphology was rounded, and by 3 days the cells were clustered. The clusters aggregated to large clumps by 7 days. In contrast, on the etched PU, cell morphology began changing to fusiform within 2 h, whereas cells on TCPS were a mixture of rounded and fusiform morphologies. Latency to confluence on etched PU was 3 days, similar to as on TCPS.

Utilizing the cyclic uniaxial tensile strain function of the bioreactor, two rounds of stimulation at supra-physiological magnitude and physiological frequency (1%, 1500 cycles at 30 cycles per min) prompted a Gaussian distribution of alignment perpendicular to the principal strain axis that was evident at 24 h [ Fig. 13(c)] but not 6 h [ Fig. 13(b)] after the final strain application. This reaction, termed stress avoidance, minimises the tensile strain transmitted to the cells^38^ that originate from a nominally compressive environment. This indicates that the substrate transmits strain to the cells. Furthermore, cells remained viable after 7 days culture in the bioreactor, demonstrating the feasibility for longer studies. Under different loading regimes, application of cyclic uniaxial strain to bone cells has been shown to induce parallel alignment (9%, 1 Hz, 8 h),^39^ and 55° alignment (8%, 1 Hz, 24 h).^40^ Physiological magnitude strain (0.25%) elicited increased extracellular matrix production but did not induce alignment in this case.^41^

## CONCLUSIONS

A polycarbonate urethane has been optimized by surface modification to improve cell adhesion *in vitro*. Plasma etching in an oxygen atmosphere significantly increased the surface hydrophilicity and surface-bound oxygen, and changed the topography. Surface roughness was reduced by etching at 20 W, and increased by etching at higher powers. Following etching at any combination of the tested parameters, the topography was changed from a segmented to an amorphous structure. Plasma etching parameters of 20 W and 40 s were selected for cell culture. The combined effect of pre-incubation of the membrane with culture medium, in addition to plasma etching, was proven to support cell adhesion and showed cell morphology similar to that on TCPS. The optimized substrate permitted cyclic uniaxial mechanical stimulation of the attached cells in the novel bioreactor as indicated by the orientation of cells to reduce stress. This indicates that the membrane is effective at transferring strain applied by the novel bioreactor.
