# Supplementary material for: Development of an elastic cell culture substrate for a novel uniaxial tensile strain bioreactor
Source: J Biomed Mater Res A. 2013 Aug 31;102(7):2356–64. doi: 10.1002/jbm.a.34917 (PMC4255296; doi:10.1002/jbm.a.34917)
Supplement: Supplementary file 1 — Supplementary Information [file jbma0102-2356-sd1.docx]

## Supplementary Data

The contact angle data for PU membranes, post-manufacture and after wetting, is shown in Figure SD 1. The wetted PU was washed with PBS and then 70 % ethanol, as was carried out for bioreactor membranes.

The XPS survey spectra for PU membranes are shown in Figure SD 2. To exclude the possibility of contamination with silicon-species occurring during processing, a membrane was prepared and cured in a vitreous carbon crucible using the same solvent processing. This sample was also positive for silicon (Figure SD 3).

XPS spectra for the low binding energy regions are shown in Figure SD 4 for samples incubated with culture media. The etched samples supported better adhesion of serum proteins, which is evident from the greater at.% of sulphur and the masking of the silicon signal from the underlying PU. The valence band showed signals for Na 2p, O 2s and O 2p, S 3s and S 3p.
